# Fatty acid secretion by the white-rot fungus, *Trametes versicolor*

**DOI:** 10.1093/jimb/kuab083

**Published:** 2021-11-12

**Authors:** Guyu Hao, Guy C Barker

**Affiliations:** School of Life Sciences, University of Warwick, Coventry, CV4 7AL, UK; School of Life Sciences, University of Warwick, Coventry, CV4 7AL, UK

**Keywords:** Fatty acids, Fungal oil secretion, Fungal biomass, *Trametes versicolor*

## Abstract

Fungi can acquire and store nutrients through decomposing and converting organic matter into fatty acids. This research demonstrates for the first time that the white-rot fungus *Trametes versicolor* has the ability to secrete extracellular droplets which can contain a high concentration of long-chain fatty acids and unsaturated fatty acids as well as monosaccharides and polysaccharides. The concentration and composition of the fatty acids varied according to the age of the droplet and the feedstock used for growth of the fungi. The results raise the possibility that these droplets could be harvested offering a new approach for the microbial generation of oil from waste.

## Introduction

There is an increased desire by society to move away from fossil-based fuels due to our rising awareness of their carbon footprint and also in an attempt to make our fuels more sustainability. The consequence is that there is a demand for novel sources of oils. Some oils are also nutritionally important due to their requirement in a healthy diet including long-chain omega-3 and omega-6 fatty acids which have functions that help prevent heart disease and stroke (Ríos-Covián et al., 2016). Among them the omega-3 polyunsaturated fatty acids (PUFA) including eicosapentaenoic acid (EPA, 20:5n3) and docosahexaenoic acid (DHA, 22:6n3) in particular have been found to be important for human health (Swanson et al., 2012), including playing an important role in prevention of diseases and control of inflammation (Dunstan et al., 2007; Serhan et al., 2008). Antioxidant properties of omega-3 fatty acids have been shown to inhibit the growth of microorganisms and (PUFAs) are efficient at preventing inflammation induced by bacterial infection (Chanda et al., 2018; Cheng et al., 2015). For example, EPA and DHA show significant antibacterial effects to against *Propionibacterium acnes* and *Staphylococcus aureus* (Desbois & Lawlor, 2013). Cheng et al., found that by editing and adding delta-6 desaturase and elongase genes (Fadsd6 and Elvol5a) the self-production of EPA and DHA was improved in transgenic zebrafish and these had enhanced resistant to *Vibrio vulnificus* infection by diminishing the attendant inflammatory response (Cheng et al., 2015). Chanda et al., also found that defensive cytotoxins could be suppressed by microbial self-synthesised PUFAs (Chanda et al., 2018). The 2015–2020 Dietary Guidelines for Americans recognises the importance of these oils and suggests a daily intake of 250 mg omega-3 PUFAs acids (Zhang *et al.*, 2018), however, conversion of EPA within the human body is only 0.3% and for DHA it is less than 0.01% (Goyens et al., 2005; Hussein et al., 2005). These fatty acids therefore need to be acquired through our diet. EPA and DHA are found in sea grasses and algae but are often harvested from sea fish where they have been accumulated. In the long term the sustainability of such fish oils is questionable and alternative sources of these PUFAs acids is needed.

Fungi acquire nutrients through decomposing and converting either dead or living organic matter into substrates for the fungi to use including fatty acids. Fungi that can break down lignin and cellulose have three specific characteristics; the coordinated growth of mycelial networks, specialised enzymes produced to attack components of wood and the ability to scavenge and redistribute phosphorus and nitrogen compounds (Watkinson, 2001). Fungi are heterotrophic organisms that require sources of organic matter for growth (Alexopoulos et al., 1996). Fungi are remarkable in their ability to undergo changes in their morphology, physiological state or behaviour in response to external environmental changes, such as temperature, moisture content, nutrition supplement and proximity to other species of fungi. They can also store resources as single cell oils (SCOs) that can be stored inside the cell, the oleaginous fungi can accumulate between 20% and 80% of their biomass as SCOs (Cohen & Ratledge, 2005). The ability of fungi to produce and store fatty acids is well known and their ability to produce fatty acids has proved beneficial with some fungi being grown for their oil which is utilised in infant formulas and cosmeceuticals (Cohen & Ratledge, 2005). Fungi species used in the production of such cosmeceuticals include *Agaricus subrufescens* and *Ganoderma lucidum*, whose oils are used for skin care and have been described to have antioxidants and skin revitalising functions. Other fungi species used for commercial purposes include *Trametes versicolor* and *Schizophyllum commune* due to their abilities to synthesise unusual fatty acids (Hyde et al., 2010; Yokoyama et al., 1975). *T. versicolor* is also known to have anticancer properties (Habtemariam, 2019). This has been attributed to the activity of *T. versicolor*-specific polysaccharides that inhibit cell growth and cause apoptosis in cancer cells and these have been approved as medicines and as adjuvants to conventional chemo- or radiation cancer therapy (Habtemariam, 2020).

*T. versicolor*, also called *Coriolus versicolor* or Turkey tail fungus, belongs to the basidiomycetes the group that include the white-rot fungi (Saleh et al., 2017). *T. versicolor* has a wide global distribution and is commonly found on dead wood or fallen trees. It has a strong ability for wood decomposition through secretion of ligninolytic enzymes to degrade lignin (Collins & Dobson, 1997). Researchers have found it produces several types of these ligninolytic enzymes that are involved in the wood degrading process such as lignin peroxidases, manganese peroxidases, and laccases (Floudas et al., 2012; Iqbal et al., 2011). Lipids and associated enzymes have also been associated with enhance degradation of lignocelllosic biomass which in *Ceriporiopsis subvermispora* has been linked to the expansion of the number of desaturase-encoding genes putatively involved in fatty acid metabolism (Fernandez-Fueyo et al., 2012). Lipid compounds have an important role in the fungal life cycle with SCO accumulation occurring predominantly in the stationary growth phase when nitrogen or phosphor availability is limited but carbon is freely available (Fagone & Jackowski, 2009). The lipids in basidiomycota are thought mainly to supply carbon and energy for cell components ensuring necessary metabolic activity and the formation and development of cell walls and organelles (Altschul et al., 1990). The production of oils in these fungi remains poorly studied. This study was designed to determine how oil production is controlled in *T. versicolor* through the use of differing substrates and conditions.

## Materials and Methods

### Microorganisms Identification

#### Fungal strains selection

*T. versicolor* samples were ordered from the Westerdijk Fungal Biodiversity Institute (Mycobank reference 281 625) and stored in the dark at 4°C.

#### DNA extraction

The fungal species were transferred to Malt Extract Agar (MEA, Sigma, 70145) plates to achieve sufficient growth for DNA extraction. 500 mg of fungal mycelium was collected and removed using metal tweezers, which were sterilised by heating in a flame for 10 s and placed into tube. The DNA extraction process followed the FastDNA^TM^ Spin Kit for soil protocol and the concentration of DNA was measured by Implen Nanophotometer^®^.

#### PCR and primer selection

Primers were designed based on the ITS1 and ITS2 region within the fungal genome (Porras-Alfaro et al., 2014), which is specific to fungal DNA and used in many fungal DNA identification before. ITS1F (5′-CTTGGTCATTTAGAGGAAGTAA-3′) and ITS4 (5′-TCCTCCGCTTATTGATATGC-3′) were ordered from Sigma-Aldrich. Lyophilised primers were mixed with sterile water and vortexed for 30 s to ensure fully mixed. Concentration of original primers was approximately 1000 μg/μl and then primers were diluted further to 100 μg/μl.

20 μl of PCR mix was prepared and added to 0.5 ml PCR tubes to amplify products from the fungal DNA. The protocol was as followed: 10 μl of REDTaq^®^ReadyMix^TM^, 3 μl of DNA template, 1 μl of forward primer (ITS1F), and 1 μl of reverse primer (ITS4) with 5 μl of PCR water (Sigma, W1754). The concentration of DNA was adjusted to between 1 μg/μl and 10 μg/μl. PCR tubes were placed in GeneAmp 9700 Thermocycler with a touchdown programme used to amplify the fungal DNA. This used 94°C for 3 min, then annealing for 1 min. The annealing temperature started at 60°C but was decreased 1°C after each cycle for 6 cycles. After that, annealing was carried out at 57°C for 1 min for 30 cycles. Extension was carried out at 72°C for 1 mine for all cycles.

#### Gel electrophoresis and DNA sequencing

Products were analysed using a 1% agarose gel and sized using a 100 bp DNA ladder (New England BioLabs, B7025). Clean PCR products were sequenced using sanger sequencing (GATC). The sequence data were subsequently analysed using NCBI BLAST (Altschul et al., 1990).

### Substrate preparation

#### Rye grain

Rye grain was used to initiate growth of the fungal stocks. 1 kg of rye grain was poured into a metal bucket and an equal volume of sterilised water added. The metal bucket was heated until the water came to the boil. The heat was turned off and stirring continued for 60 min allowing the grain to soften. Surplus water was then drained and 150 g of rye grain dispensed into individual glass honey jar. Jars containing rye grain were sterilised at 123°C for 60 min then this autoclaving repeated to ensure sterility. When the jars were sterilised and cooled down, sterilised tweezer was used to transfer fungus sample to the new jars while under a sterile laminar flow hood. Inoculated jars were incubated at 20–30°C.

#### Straw

Wheat straw was provided from the Wellesbourne campus, Warwick Crop Centre. Straw was blended using a food processer for 5 min to chop the straw into 0.5–1 cm small pieces. 10 g straw was added to each glass jar and these were then autoclaved using two cycles of 123°C for 60 min. Before incubating the fungus, 60 ml sterilised water was added to ensure the optimal growth conditions. After inoculation, the jar was closed and sealed by parafilm to prevent contamination and to prevent moisture loss.

### Fungal Oil Extraction and Composition Analysis

#### Fungal oil extraction and transesterification

50 μl of fungal oil or 200 mg fungal mycelia (for fungus growth on agar) was transferred into a vial with 500 μl of 1 N MeOH/HCl. 10 μl of 25 mg/ml 15:0 free fatty acid (FFA) standard was added to each screw-cap vial (Supelco^®^ Sigma, 27 079). The FFA standard was prepared by dissolving 0.5 g tripentadecanoin (Sigma, T4257) in 20 ml of HPLC-grade Chloroform (Rathburn, RH1009). The tubes were vortexed to mix and incubated at 80°C for 24 hr to ensure complete derivatisation. After 24 hr, vials were left to cool to room temperature. 250 μl of 0.9% KCl and 250 μl of HPLC-grade Hexane (Rathburn, RH1002) were then added and the layers allowed to separate. The upper hexane layer was then transferred to a new vial, for analysis using gas chromatography.

#### Gas chromatography analysis

An Agilent Technologies 6850 GC/MSD system with 10 μl 701N-CTC-26s/AS needle (P/N, 203205) was used to analyse the samples. Nitrogen, hydrogen, and air flow taps were switched on to allow the air flow within the column (BPX70). Constant flow of the nitrogen was kept at 30 ml/min and the Detector Flame Ionisation Detector was set temperature to reach 240°C. The injection needle was washed using HPLC-grade hexane between each sample loading. 1 μl of hexane was used as a negative control and 1 μl of 10 mg/ml component FAME Mix (Supelco^®^37, CRM47885) was used as an analytical standard for calibration. These were run prior to samples being analysed, following analysis, all data were compiled, and results reported as the amount of each fatty acid.

### Monosaccharide Analysis

#### Hydrolysis of polysaccharide

10 μl of secreted fungal sample was mixed with 1 mL trifluoroacetic acid (TFA) in a 2 mL eppendorf tube that was sealed under a nitrogen atmosphere. Hydrolysis of the polysaccharide was carried out as follows; neutral sugars 6 hr, 120°C and amino sugars 3 hr, 100°C (Guo et al., 2019). After cooling to room temperature the sample was evaporated to dryness under reduced pressure. The dried sample was then used for the following experiments.

#### Derivatisation of monosaccharide and hydrolysed polysaccharide

The hydrolysed polysaccharides were dissolved in 100 μl 28–30% ammonia solution. A further 10 μl of un treated fungal liquid was dissolved in 100 μl 28–30% ammonia solution, 100 μl of this solution was mixed with 100 μl 0.5 mol/L methanolic solution of reagent PMP (1-phenyl-3-methyl-5-pyrazolone) in a 2 ml eppendorf tube that was sealed under a nitrogen atmosphere. The mixture was allowed to react for 30 min at 70°C in water bath, then cooled to room temperature and then concentrated for about 1.5 hr to dryness under nitrogen. 1 ml water and 1 ml chloroform were added and the tube shaken until the sample was completely dissolved. The aqueous layer was carefully removed and filtered through a 0.2 μm filter prior to LCMS analysis.

#### LC-MS conditions

A Bruker Amazon ETD ion trap coupled with Dionex 3000RS UHPLC and mass spectrometer equipped with an electrospray source in positive ion mode (MS range 50–2000 m/z) was used with an Agilent Zorbax Eclipse C18, 100 × 2.1 mm, 1.8 um column and the following gradient mobile phases. A: 25 mM ammonium acetate in water; B: acetonitrile with 0.1% formic acid. The Gradient was as follows, 0–5 min, 15% B; 5–25 min, 50% B; 25–30 min, 100% B; 30–35 min, 100% B; 35–40 min, 15% B; then isocratic for 17 min before next injection. The flow rate was 0.2 m/min and the injection vol was 5 ul.

## Results

**Fig. 1.**
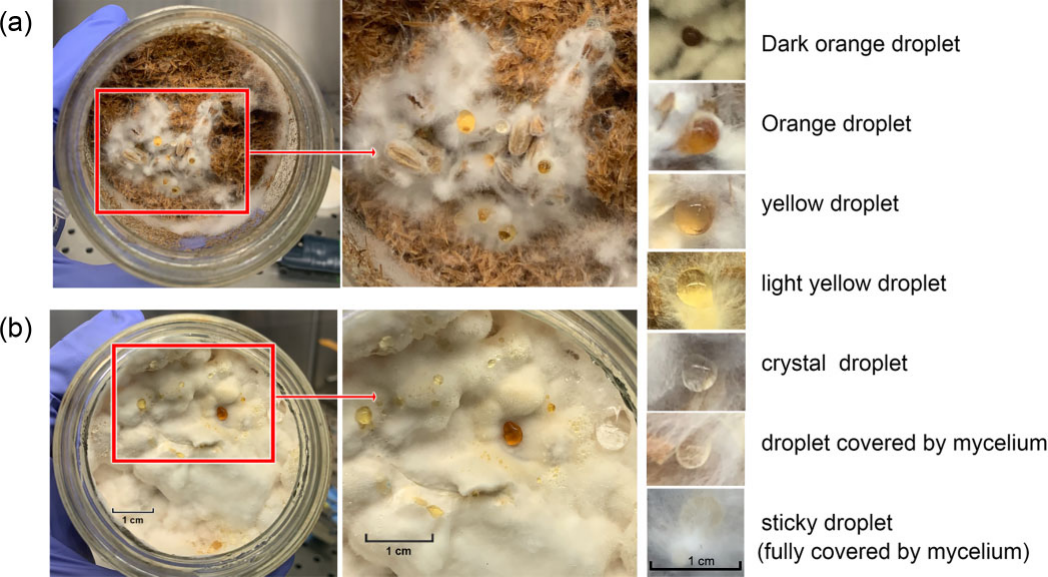
*T. versicolor* growth on straw-based substrate (a) and rye grain-based substrate (b). Honey jars contained 10 g sterilized straw with 60 ml water or 150 g prepared rye. The jars were incubated for 24 hr after inoculation with infected rye grains. The image in the middle is a 2× magnification of the image on the left. The smaller images illustrate the different appearance and texture of the droplets at 10× magnification.

Optimal cultivation of *T. versicolor* on either straw or rye optimal growth was achieved following the addition of 60 ml water. The optimal temperature was found to be 24–26°C. During the cultivation liquid “droplets” were observed on the surface of mycelium (Fig. 1). These appeared to be secreted by *T. versicolor* when either substrate was used. These droplets could be separated, based on their colour and appearance, into three groups. Dark orange, yellow or light-yellow, clear crystal or sticky texture droplets.

**Fig. 2.**
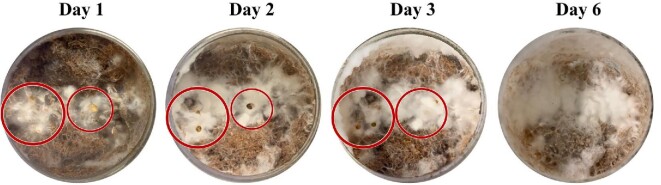
Droplets secreted by *T. versicolor* on straw at day 1, day 2, day 3, and day 6. The moisture content of substrate is 6 ml water per gram straw. Every day after fungus produced oil drops, drops were removed. At day 3, oil drops were allowed to remain at the place.

To explore the reason that different drops produced by the fungus had different appearances, the process from the oil drop appearance to its end as the oil drop disappears was recorded (Fig. 2). As the droplets are first formed, they are small and dark orange, with a volume of around 3–5 μl. Over time, the drops grow, while their colour lightens to yellow. These yellow drops have an average volume of 10–20 μl while a few swelled to 50 μl. On further incubation, the drops appearance becomes sticky, and the surface is covered by fungal mycelium. A short time after this the drops disappears and there appears to be rapid mycelium growth at the location. The process was observed to occur over a 24 hr period, but it can be more rapid with the drops disappearing within 6 hr.

If the drop was removed at an early stage, it was observed that within a day a fresh drop was produced by the fungus in the same location or adjacent. If the drops are not removed, then the fungus stops producing drops as seen in Fig. 2. When drops stopped being removed at day 3 by day 6 no more droplets were seen. Based on the above phenomenon, it was conjectured that if oil drops are removed daily without interruption the fungus could potentially continuously produce oil drops. To prove this 20 jars of *T. versicolor* grown on straw were observed until the fungus started to produce drops. The drops were then removed daily with results recorded for 22 days. Drops were observed and recorded for the whole period. The growth rate of the fungus was also observed to significantly decrease when the drops were continuously removed.

In the first 7 days, a total of 50 ul of dark droplets can be produced, with a total fatty acid content of about 30–40 mg, average of 3–4 mg of fatty acids per 1 g of straw. After that, droplets will continue to be produced, but the fatty acid content is significantly reduced, and are replaced by other components.

These droplets they were subsequently analysed for the presence of fatty acids and for monosaccharides in order to determine their origin.

**Fig. 3.**
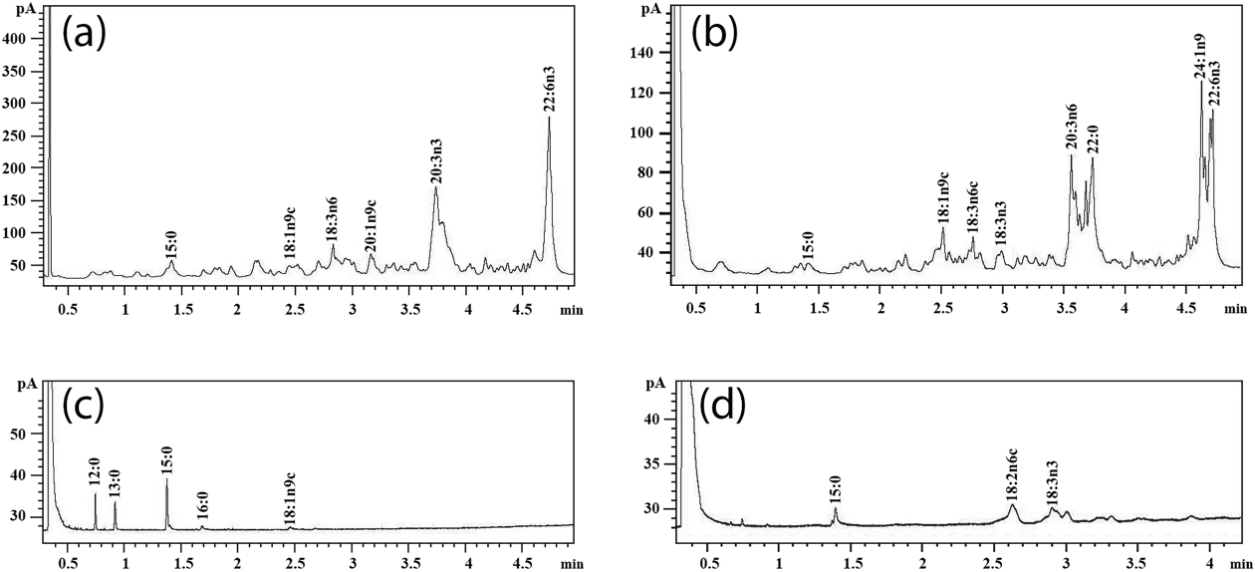
GC analysis of fatty acid composition of “dark orange” and “light yellow” colour droplets mixture from rye grain-based substrate and straw-based substrate. These droplets were harvested form *T. versicolor* cultures grown on straw (6ml water per gram straw) or prepared rye grain. The average volume of each drop was 3–5 μl, several droplets were therefore combined to give the total volume of 50 ul required for subsequent analysis. A fatty acid 15:0 standard at a concentration of 5000 ng/μl was used to ensure the transesterification protocol is consistent. (a) Dark orange colour droplets mixture from rye grain-based substrate. (b) Dark orange colour droplets mixture from straw-based substrate. (c) Liquid from the bottom of the rye grain-based substrate jars. (d) Light yellow droplets mixture from straw-based substrate.

Fig. 3 shows the composition and concentration of fatty acids within the different droplets secreted by *T. versicolor*. Table 1 shows the concentration of the transesterified fatty acids in different droplets harvested and Table 2 gives a percentage of SFA, MUFA, and PUFA in different fungal secretion droplets from the two substrates. Compared with the *T. versicolor* fungi grown on straw, that grown on rye grain produced smaller and darker oil droplets and these took longer to form. The droplets collected from the rye grain (Fig. 3a, one sample result) gave a maximal total concentration of fatty acids of 255.43 ug/μl. The principle fatty acids being eicosatrienoic acid (20:3n6, 14.57 ug/μl, behenic acid (22:0; 7.40 ug/μl), nervonic acid (24:1n9, 6.04 ug/μl) and docosahexaenoic acid (DHA) (22:6n3; 34.21 ug/μl). In comparison the drops collected from the rye-based substrate had higher concentration of very long-chain fatty acids, with aliphatic tails of 22 or more carbons. Other fatty acids also were also found in a high concentration, including oleic acid (18:1n9; 11.762 ug/μl), linolenic acid (18:3n6; 14.26 ug/μl). Medium chain fatty acid (10:0, 11:0, 12:0, 13:0, 14:1n5) and a small amount of short-chain fatty acids (8:0) were found in rye grain-based substrate liquid and in straw cultures (Fig. 3c), the reason for this phenomenon needs to be explored in further work.

**Table 1.** The Concentration of Fatty Acids in Secretion Droplets

(A)	Straw orange (*n* = 5)		Straw yellow (*n* = 8)		Straw white (*n* = 11)	
Fatty acid	Average	SD	Average	SD	Average	SD
**16:0**	0.12	0.27	1.08	0.93		
**16:1n7**	0.89	1.67				
**17:0**	2.34	5.16				
**17:1n7**	1.58	2.79				
**18:0**	2.17	4.00				
**18:1n9c**	0.25	0.35	0.19	0.53	2.92	4.65
**18:2n6t**	3.47	7.37	0.31	1.03		
**18:2n6c**	2.09	2.14	2.52	4.04	0.26	0.37
**18:3n6**	8.56	8.22				
**18:3n3**	0.52	0.44	1.15	3.25		
**20:0**	4.87	4.46	0.50	1.41		
**20:1n9**	3.15	2.39				
**20:2n6 + 21:0**	2.26	1.48				
**20:3n6**	10.84	6.67				
**20:4n6**	0.98	1.10				
**20:3n3**	3.27	1.68				
**22:0**	4.87	3.84				
**22:1n9**	3.30	4.83				
**20:5n3**	1.98	1.48				
**23:0 + 22:2n6**	5.16	8.03				
**24:0**	3.22	2.63				
**24:1n9**	4.18	3.46				
**22:6n3**	2.31	1.00				
**(B)**	**Rye orange (*n* = 7)**		**Rye aqueous solution (*n* = 5)**			
**Fatty acid**	**Average**	**SD**	**Average**	**SD**		
**11:0**	0.00	0.00	0.94	1.89		
**12:0**	8.19	21.25				
**13:0**	2.57	4.70	2.25	1.03		
**14:0**	0.80	2.11				
**14:1n5**	0.91	1.70				
**15:1n5**	2.21	2.67				
**16:0**	1.64	2.01	0.35	0.33		
**16:1n7**	1.33	1.83				
**17:0**	14.77	31.94				
**17:1n7**	11.22	22.55				
**18:0**	34.27	57.87				
**18:1n9t**	13.08	32.47				
**18:1n9c**	10.17	17.96				
**18:2n6t**	3.63	4.08				
**18:2n6c**	4.90	6.24				
**18:3n6**	10.48	12.11				
**18:3n3**	6.64	8.41				
**20:0**	2.67	3.73				
**20:1n9**	6.06	8.87				
**20:2n6 + 21:0**	6.82	6.71				
**20:3n6**	34.58	30.82				
**20:4n6**	5.84	4.61				
**20:3n3**	15.94	13.59				
**22:0**	15.12	19.06				
**22:1n9**	4.23	7.72				
**20:5n3**	4.96	5.92				
**23:0 + 22:2n6**	9.97	15.55				
**24:0**	4.35	4.00				
**24:1n9**	4.29	2.57				
**22:6n3**	17.42	16.09				

Droplets were harvested form *T. versicolor* cultures grown on straw (6 ml water per gram straw) or prepared rye grain. The average volume of each droplet was 3–5 μl, in order to provide the 50 ul required for subsequent analysis 10 were collected and combined. Concentrations are indicated as (g/L). The percentages represent the different groups of fatty acids as a fraction of the total fatty acid (total FA) measured for a given sample. *n* = number of samples for Fungal Rye Grain-Based Substrate (*n* = 7) and Straw-Based Substrate (*n* = 5). SD represents the standard deviation obtained from the different repeats. (A): *T. versicolor* growth on straw-based substrate; (B): *T. versicolor* growth on rye grain-based substrate.

**Table 2.** The Total Concentration and Percentage of Saturated Fatty Acids (SFA), Monounsaturated Fatty Acids (MUFA) and Polyun-saturated Fatty Acids (PUFA) in Fungal Secretion Droplets Mixture Obtained from Rye Grain-Based Substrate (*n* = 7) and Straw-Based Substrate (*n* = 5)

	Straw orange	%	Rye orange	%
**Total FA**	73.33		259.24	
**SFA**	17.77	24.23	84.56	32.62
**MUFA**	14.14	19.28	53.50	20.64
**PUFA**	41.42	56.49	121.18	46.74

The average volume of each drop was 3–5 μl, several droplets were therefore combined to give the total volume of 50 ul required for subsequent analysis. Percentages represent the fraction of total fatty acid (total FA) measured for a given sample.

The droplets collected from the straw-based substrate (Fig. 3b, one sample result) yielded a maximum total concentration of fatty acids to 159.748 ug/μl. The concentration of DHA (22:6n3) was 1.61 ug/μl and nervonic acid (24:1n9) was 3.75 ug/μl. In addition to these long-chain fatty acid end products, a range of concentrations of a variety of other fatty acids which could be their precursors were also found in the drops. For example, eicosatrienoic acid (20:3n6, 20:3n3) and docosadienoic acid (22:2n6) are intermediate products in the biosynthetic pathway, which respectively reach to 54.37 ug/μl, 23.74 ug/μl and 18.92 ug/μl. The concentration of arachidonic acid (20:4n6) was 4.94 ug/μl, this can be converted by eicosatrienoic acid (20:3n6) using △5-desaturase directly. Erucic acids (22:1n9) can also act as an immediate precursor of nervonic acid (24:1n9), the concentration of this reached to 1.16 ug/μl.

**Fig. 4.**
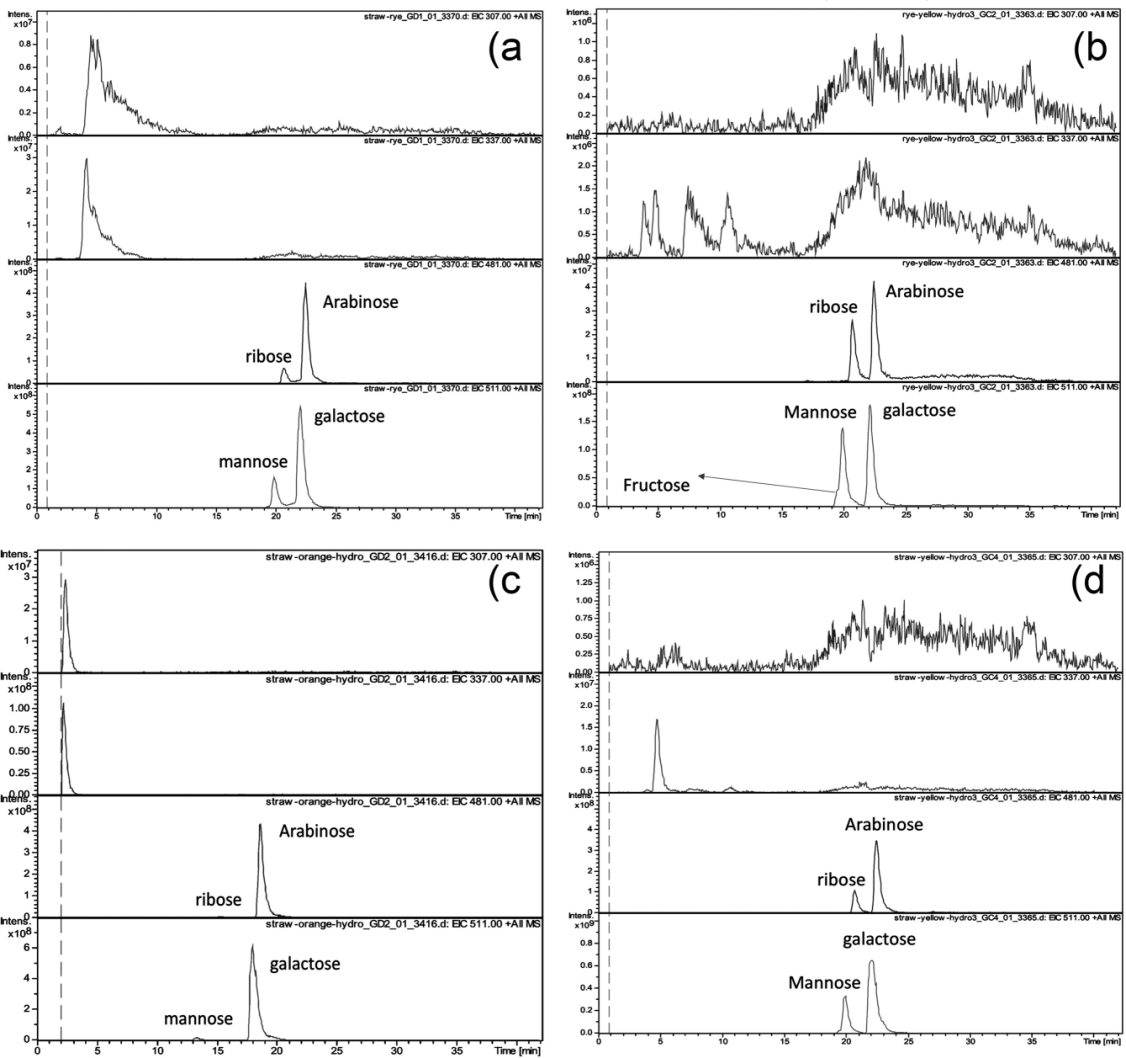
LCMS monosaccharide analysis of fungal-secreted “orange” droplets and “yellow/white” colour droplets mixture obtained from rye grain-based substrate and straw-based substrate. The different panels are (a) orange colour droplets mixture from rye grain-based substrate, (b) yellow colour droplets mixture from rye grain-based substrate, (c) orange colour droplets mixture from straw-based substrate, and (d) yellow/white colour droplets mixture from straw-based substrate. The peaks are determined from alignment against reference standards. The peaks showing multiple peaks with no clear label indicate the presence of other sugars.

As well as being analysed for fatty acids the droplets were analysed for monosaccharide sugars. As indicated in Fig. 4, key monosaccharides were identified in the fungal-secreted aqueous droplet including fructose, glucose, galactose, mannose, arabinose, ribose and xylose. The majority of the monosaccharides were arabinose, ribose, galactose, and mannose. The amounts and diversity of monosaccharides increased as the droplets colour changed. Initially in the orange droplets small amounts of ribose and mannose were detected however, in the clear droplets these monosaccharides had become the principal components.

## Discussion

This study demonstrates that the white-rot fungi, *T. versicolor* has the ability to secrete extracellular droplets. It is widely recognised that fungi secrete acids and digestive enzymes to access surrounding nutrition sources and break these down into simple molecules for absorption. The production of SCOs by fungi relies on the availability of certain nutrients from the feedstock on which the fungi is feeding. It has been suggested that oil production increases in the presence of excess carbon source and limited nitrogen. When the carbon source and nitrogen are balanced, all nutrients are provided to the cells to encourage the growth. Once nitrogen is exhausted and the fungus continues to assimilate carbon source, such as glucose, glucose will enter to the lipid biosynthesis pathway causing lipid accumulation (Ratledge, 2013). However, the exact reason for oil production in fungi is unclear as it often unstable being produced early in growth and then utilised. Research has proved that nitrogen supplementation alters the growth of fungi and can significantly effect the lipid composition produced by fungi (Madani et al., 2017). Other studies confirmed that nitrogen starvation activates and limitation of other nutrients can influence the quality of lipids (Tudzynski, 2014).

In addition to their important role as constituents of biological membranes, lipids can be stored in distinct organelles (Rego et al., 2014). However, only minor amounts of lipids are found in lipid-synthesising organelles, such as the endoplasmic reticulum (ER), the Golgi apparatus, and mitochondria (Fagone & Jackowski, 2009; Tamura et al., 2013). Excess cellular lipids can also induce lipotoxicity which is harmful for the cell, lipids are therefore translocated from synthetical site to specialised organelles and converted to storage lipids (Klug & Daum, 2014). Those can subsequently be used for producing energy or synthesis of complex lipids (Rajakumari et al., 2008). Such movement of lipid requires specific lipases that can catalyse hydrolysis of hydrophobic polymers into free fatty acids, which can be transported using active transport mechanisms, and when a high concentration of free fatty acids occurs they freely diffuse into cells (Fickers et al., 2005; Kohlwein & Paltauf, 1984). Although the principles of process of fungal fatty acids absorption and transportation between cells have not been elucidated, observation have led to the hypothesis that uptake of alkanes by cells is a passive, diffusion-like process (Fickers et al., 2005; Fukui & Tanaka, 1981). In the fungal catabolic pathways, free fatty acids derived from long-chain alkanes (C14-C18) can be directly used for lipid synthesis. This process involved partial elongation and desaturation results long-chain saturated and unsaturated fatty acids (C16–C18) are incorporated into cellular phospholipids for transport and storage (Fickers et al., 2005).

Unlike nonoleaginous organisms that store lipids within the cell, secretion of SCOs in oleaginous organisms is not commonly recognised. The only reference found which had some reference was in the fungus *Mortierella alpina* that normally accumulates lipid in the hyphae but small lipid droplets were detected on the surface of some mycelia (Wang et al., 2011). It is well known that different fungal species can produce oil (Blümke et al., 2014) but it has been assumed that this oil is not secreted. The production of the droplets we observed was rapid (as early as 6–12 hr) however any droplet if undisturbed was only present for a short period (2–3 days) and their subsequent reabsorption indicates a specific role. Long-chain poly unsaturated fatty acid, monosaccharides, and polysaccharides were also detected within these droplets. The fatty acid composition of the droplets was found to differ depending on the substrate on which the fungi were grown indicating that the nutrient content of the substrate may influence the concentration of fatty acid that is contained within the droplet.

The effect of stress on the growth of *T. versicolor* was also tested by changing the growth conditions and altering the temperature. The optimum growth temperature of *T. versicolor* was found to be between 25°C and 30°C and growth is rapidly suppressed at temperatures ranging above 30°C and below 20°C (Jo et al., 2010; Xiaoyu et al., 2013). The moisture content of the solid substrate is an important external environment for fungi growth. Suitable moisture conditions allow formation of sufficient films which covers the substrate while allowing air flow to circulate. The optimal moisture content of the substrate does differ depending on the fungus and can effect growth (Reid, 1985; Zadražil & Brunnert, 1982), droplet formation was only observed under optimal conditions.

Fig. 3 shows that the concentration of fatty acid inside the older yellow drops was significantly reduced compared with that found in the orange drops and that the fatty acids 18:3n3 and 18:2n6 had become the predominant fatty acids. The secretion of yellow droplets was observed up to 3 months from the initial inoculation of the culture. These yellow drops were larger than the orange droplets but the concentration of fatty acids and the chain lengths of the fatty acids present within them was less. This might suggest that the yellow droplets might be used for more basic nutrient storage compared to the orange droplets. These droplets were determined to be capable of promoting fungal growth as when the droplets were transferred to a new culture the growth rate of that culture was substantially increased. It is therefore postulated that the fungus produces such drops as a temporary energy store to enable rapid growth when required. This could explain that why fungus preferentially produced the droplets during the early growth stage. Yeast cells are well known to take up exogenous fatty acids for subsequent rapid incorporation into glycerolipids (Kohlwein & Paltauf, 1984) and these lipid stores might also act to facilitate rapid growth.

Although the reason for fungal extracellular oil droplets secretion has yet to be confirmed, it is our hypothesis that at the fungal initial growth stage, the speed of digestion exceeds the speed of the fungi reproduction. The consequence is insufficient space for storage *in vivo*, consequently, the surplus is secreted *in vitro* as a means of storage and to reduce toxicity. As the fungus multiplies there is sufficient storage *in vivo* and the extracorporeal fatty acids are reabsorbed to provide an accessible carbon source/nutrition for further growth and reproduction. The reason why the fungi should produce long-chain and highly unsaturated fatty acids stills needs to be clarified. One possibility is that they confer protection against competitors. In one experiment in which the *T. versicolor* culture was contaminated with another fungus it rapidly overgrew the competitor and droplets were observed in the fungal succession area. This was repeated in further experiment that were deliberately inoculated with other fungi (Pers Comm). There is some possibility that the long-chain fatty acids could act as antibacterial/antifungal substances to improve resource competitiveness. Short-chain and long-chain fatty acids are known to have potent antifungal effects (Altieri et al., 2009; Fischer et al., 2014; Frank et al., 2018), including 6:0, 11:0, 13:0, myristic (14:0), palmitoleic (16:1), oleic (18:1n9), and linoleic (C18:2n6) acids that have shown significantly stronger activity against fungi (Frank et al., 2016; Gołębiowski et al., 2014). PUFAs have also been proved to have antimicrobial potency and antiinflammatory properties and have more effectively inhibition ability than unsaturated long-chain fatty acids (Chanda et al., 2018; Desbois & Lawlor, 2013). In addition, monoacylglycerols have been shown to inhibit fungal growth (Altieri et al., 2009; Frank et al., 2018). Another possible role is to aid lignin breakdown. The white-rot fungus *C. subvermispora* produces large amounts of linoleic acid (18:2n6) during the early stage of wood decay and degrades lignin by manganese peroxidase-catalysed lipid peroxidation. It shows a relationship between fatty acid metabolism and selective lignin degradation (Watanabe et al., 2010). It is possible that in *T. versicolor* these lipids also act to facilitate lignin degradation a role which could prove important in helping us understand biodegradation by lipid radicals.

Although the function of these fatty acid droplets has yet to be demonstrated, we show that *T. versicolor* can both synthesize and secrete fatty acids. It is known that fungi can accumulate a great quantity of SCOs (Madani et al., 2017; Sakuradani, 2010), and these and other products are already widely utilised in biotechnology (Cohen & Ratledge, 2005; Rivaldi et al., 2017). The detection of unusual fatty acids, in particular the highly polyunsaturated long-chain fatty acids, within the secreted droplets raise the possibility that these could be harvested for use in the production of higher value oils that could be used to replace current fish oil supplements. The results from this study show that given the right feedstocks and growth conditions *T. versicolor* could be used for the production of these nutritionally important oils and harvesting could be vastly simplified compared to current approaches. The detection of polysaccharides as well as oil within the droplets also suggests that the droplets could prove important as a source of compounds known to have anticancer properties (Saleh et al., 2017).

## Conclusion

This research demonstrates that the white-rot fungus *T. versicolor* has the ability to secrete extracellular droplets, which were found to contain long-chain fatty acids and unsaturated fatty acids. The composition of these fatty acids varied depending on the age of the droplet and the feedstock used for growth of the fungi. Our findings would suggest that the mycelium is synthesising fatty acids which are then transported via the mycelium and then secreted forming oil droplets. It is also observed that droplets produced by the fungus can promote fungal growth and improve resource competitiveness. Although the exact reason for the droplet formation in *Trametes versicolor* is unclear, the utility of their constituents in improving health makes further investigation important.

## Data Availability

The data underlying this article are available in the article.
